# COVID-19 Vaccination Uptake and Effectiveness for Hospitalized Cases Among Healthcare Workers in Tertiary Hospital

**DOI:** 10.3390/vaccines13020147

**Published:** 2025-01-31

**Authors:** María Eugenia Jiménez-Corona, Luis-Pablo Cruz-Hervert, María del Rocío Sánchez-Díaz, Gabriel Chavira-Trujillo, Aída Jiménez-Corona, María del Rosario Vázquez-Larios

**Affiliations:** 1 Departamento de Epidemiología, Instituto Nacional de Cardiología Ignacio Chávez, Mexico City 14080, Mexico; rociosan169@gmail.com; 2 Dirección de Prestaciones Médicas, Instituto Mexicano del Seguro Social, IMSS, Mexico City 06600, Mexico; psic.gabrielchavira@hotmail.com; 3 Dirección General de Epidemiología, Secretaría de Salud, Mexico City 01480, Mexico; aidaajc@gmail.com; 4 Laboratorio de Microbiología, Servicio de Infectología y Microbiología, Instituto Nacional de Cardiología Ignacio Chávez, Mexico City 14080, Mexico; rosariovazquez_larios@prodigy.net.mx

**Keywords:** COVID-19, healthcare workers, vaccination, hospitalization, tertiary hospital

## Abstract

**Background/Objectives**: Healthcare workers (HCWs) faced elevated risks during the coronavirus disease 2019 (COVID-19) pandemic. Vaccination among HCWs was a key strategy to mitigate severe outcomes and maintain healthcare system functionality during the crisis. The aim of this study was to assess the distribution, severity, and clinical factors associated with COVID-19 among HCWs in a tertiary hospital across eight pandemic waves and evaluate the effectiveness of vaccination in reducing severe outcomes. **Methods**: A cross-sectional study analyzed data from HCWs at a high-specialty hospital in Mexico City from March 2020 to February 2024. Sociodemographic, clinical, and vaccination data were collected and analyzed via bivariate and multivariable logistic regression to identify the factors associated with infection and severity. **Results**: A total of 7049 cases were analyzed, and 2838 (40.26%) were confirmed COVID-19 cases. Severe outcomes, including hospitalizations and deaths, were most common during the early waves, with 83.3% of severe cases occurring among unvaccinated individuals. Vaccination significantly reduced infection risk, with individuals receiving two or more doses showing a lower likelihood of infection (OR 0.67; 95% CI 0.51–0.89; *p* = 0.005). Older age; comorbidities such as hypertension and obesity; and symptoms such as fever were associated with increased severity. Compared with earlier coverage, enhanced vaccination coverage significantly lowered the hospitalization risk during the later waves (OR 11.11; 95% CI 1.2–110.2; *p* = 0.040). **Conclusions**: Vaccination effectively reduced severe COVID-19 outcomes among HCWs, demonstrating its critical role in mitigating the disease burden despite the high risk of exposure. Strategies such as targeted vaccination campaigns and continuous surveillance are essential to protect HCWs and ensure healthcare system resilience.

## 1. Introduction

The COVID-19 pandemic has profoundly impacted global health, with healthcare workers (HCWs) experiencing increased exposure risks due to their frontline roles [1,2,3,4,5]. As of March 2024, the pandemic has resulted in over 700 million infections and nearly 7 million deaths worldwide. In Mexico, the toll has been severe, with over 324,436 fatalities among 5.7 million confirmed cases over two years, underscoring the vulnerability of HCWs, who face elevated risks due to prolonged exposure to COVID-19 within healthcare settings [1,2,3,4,6,7]. Within these environments, HCWs contend with high viral loads, limited personal protective equipment (PPE) at times, and the constant demand for infection control [5,8,9,10]. These risks, paired with the essential nature of their work, make infectious disease surveillance critical for identifying and controlling infection trends in this vulnerable group [8,11,12,13]. As vaccines were developed and rolled out, healthcare workers were prioritized for vaccination in many countries, suggesting that they would serve as role models and help reduce the burden of the pandemic [14]. However, concerns have emerged about vaccine hesitancy and refusal among HCWs, which could undermine vaccination efforts and pose risks to both workers and patients [6,15,16,17]. The factors influencing hesitancy include trust issues, misinformation, personal beliefs, and demographic or professional roles, all of which contribute to lower vaccination rates and heightened vulnerability to infectious diseases [18,19,20].

The evolution of COVID-19 through multiple epidemiological waves presented a fluctuating risk landscape for HCWs, as virus variants and changing social conditions influenced transmission rates and disease severity [8,11,12,15]. Vaccination campaigns have been swiftly implemented to reduce COVID-19 morbidity and mortality, yet the protective impact of vaccination, particularly across multiple doses, requires ongoing assessment [21,22,23,24]. The literature has highlighted HCWs’ increased susceptibility to severe COVID-19, revealing demographic patterns, including age and occupation, that increase vulnerability [15,25]. Despite vaccine availability, unanswered questions remain regarding how factors such as age, comorbidities, symptomatology, and vaccination influence outcomes among HCWs during successive waves of the pandemic [3,11,15,16,26].

Several themes emerge from the literature. First, research has documented epidemiological patterns among HCWs, observing fluctuating infection rates and severities across waves and identifying the specific risk factors linked to poor outcomes [7,11,12]. Studies have also underscored that sociodemographic factors, such as age and comorbidities such as obesity, hypertension and diabetes, are significant predictors of disease severity [3,19,23,24]. Furthermore, the literature on symptomatology indicates that symptoms such as fever, cough, and fatigue are prevalent among COVID-19-positive HCWs, with certain comorbidities increasing the degree of severity [7,24,25]. The role of vaccination in mitigating severe outcomes is supported by emerging data; however, gaps persist in understanding the real-world impact of multidose vaccination schedules, particularly among HCWs who are repeatedly exposed to SARS-CoV-2 [19,22].

This study addresses these gaps by evaluating the impact of COVID-19 on HCWs in a high-risk hospital setting, considering the factors that influence the disease incidence, severity, and vaccine effectiveness across multiple waves [15,25,27]. Although prior studies have provided initial insights, comprehensive data examining the interaction effects of sociodemographic, clinical, and vaccination-related factors on COVID-19 outcomes among HCWs are limited [10,15,16,26,28]. Addressing these elements is crucial for developing effective strategies that safeguard healthcare personnel and increase hospital resilience during pandemic scenarios.

The COVID-19 pandemic has had a profound impact on healthcare workers (HCWs), yet there is limited comprehensive research analyzing the interplay among epidemiological trends, vaccination schemes, clinical characteristics, and the severity of infection within this population. Addressing this gap, the current study aims to achieve the following objectives: (1) to describe the distribution of COVID-19 cases across eight epidemiological waves, analyzing incidence, severity, and trends in suspected and confirmed cases; (2) to evaluate the sociodemographic and clinical characteristics distinguishing HCWs with and without COVID-19; (3) to identify and describe prevalent symptoms and comorbidities among confirmed cases; (4) to examine the vaccination scheme over successive doses, observing vaccine selection and completion rates; and (5) to compare the sociodemographic and clinical factors between severe (hospitalized) and non-severe cases, identifying risk factors for increased severity through multivariable analysis.

This study analyzed data from a large specialty hospital that functions as a COVID-19 hospital in Mexico City during the COVID-19 pandemic. We analyze the period between March 2020 and February 2024 and provide critical insights into the COVID-19 risk landscape for HCWs, informing strategies that enhance protective measures, optimize vaccination protocols, and guide infection control policies within healthcare settings.

## 2. Materials and Methods

This study analyzed data from the Epidemiological Surveillance Program for Healthcare Workers (HCWs) at the National Institute of Chest Diseases (INCICH), during the period from 15 March 2020 to 20 May 2024, using a structured questionnaires. This study aimed to identify and monitor respiratory symptomatic cases and contacts among HCWs, assessing the impact of sociodemographic and clinical characteristics on COVID-19 severity. The study adhered to the STROBE guidelines for cross-sectional studies, ensuring transparency in design, setting, participants, variables, data sources, and statistical analysis.

### 2.1. Study Design and Setting

We used a cross-sectional study design that was carried out at the INCICH, where a surveillance system was implemented to track respiratory symptoms and potential COVID-19 contacts among HCWs.

The healthcare workforce (HCW) at INCICH includes a diverse range of professionals, including clinical personnel such as medical staff (cardiologists, cardiovascular surgeons, and other specialists providing specialized medical care), medical residents (physicians in training undergoing practical and specialized education in cardiology), nurses (nursing professionals assisting in medical procedures and patient care), paramedics (personnel trained to provide emergency medical care and life support), medical students (individuals in academic training participating in clinical activities under supervision), and researchers dedicated to scientific research in the field of cardiology. Non-clinical personnel include administrative staff responsible for the management, administration, and operation of the institute, and general services staff in charge of maintenance, cleaning, and other essential services for the institute’s functioning. The total number of HCWs fluctuates between 2800 and 3100 individuals throughout the year. Detailed statistics about each category are provided in the results section.

Surveillance and monitoring was conducted through direct communication (WhatsApp and cellular contact) to ensure the timely identification of symptomatic individuals and their contacts. The epidemiological waves according to the definitions of Loza et al. [29] were identified as follows: the first wave spanned from 15 March 2020 to 29 September 2020. The second wave extended from 30 September 2020 to 15 April 2021. The third wave occurred from 16 April 2021 to 21 October 2021. The fourth wave lasted from 22 October 2021 to 4 March 2022. The fifth wave followed from 5 March 2022 to 19 August 2022. The sixth wave was observed from 20 August 2022 to 4 February 2023. The seventh wave extended from 5 February 2023 to 13 May 2023. Finally, the post-COVID-19 emergency period spanned from 14 May 2023 to 20 May 2024.

### 2.2. Participants

The study included HCWs at the INCICH with suspected COVID-19 symptoms, like cough, fever, and headache, or HCWs who were identified as contacts of confirmed cases. Confirmed cases were identified through RT-PCR testing, as recommended by national guidelines. The eligibility criteria included employment at the INCICH during the study period and participation in the surveillance program. Suspected patients underwent clinical assessment and completed a structured questionnaire in which sociodemographic, clinical, and exposure-related information was collected. The operational definitions of “suspected cases” and “confirmed cases” were used according to the guidelines of the Epidemiological Surveillance System of the General Directorate of Epidemiology of the Ministry of Health.

“Suspected cases” are patients seeking medical care as suspects (with symptoms or after contact with a confirmed case”. “Confirmed cases are individuals with a quantitative reverse transcription polymerase chain reaction test (RT-PCR) positive for SARS-CoV-2”.

[30]

### 2.3. Variables

The information collected included demographic characteristics (age, sex), occupational role, symptoms, comorbidities (hypertension, obesity, diabetes), need for hospitalization, status at the end of the episode, and the number of COVID-19 vaccine doses. Previous COVID-19 infections represented the number of times individuals were diagnosed with confirmed COVID-19 infections prior to the actual event. The primary outcomes were the COVID-19 positivity rate and the incidence of severe outcomes, which were defined as hospitalization or death. Severe cases and all outcomes, exposures, and potential confounders were defined prior to analysis.

### 2.4. Data Sources and Measurement

Data were collected through questionnaires administered to symptomatic individuals and contacts. Nasopharyngeal swabs for PCR testing were collected by trained personnel and processed by the Microbiology Laboratory at the INCICH to confirm COVID-19 status, according to the standardized guidelines of the Institute of Diagnostic and Epidemiological Reference (InDRE) [30]. Standardized methods were used for data collection to ensure comparability across participants. All confirmed cases were followed clinically until discharge.

### 2.5. Bias

To mitigate selection bias, active surveillance through direct communication was employed to capture symptomatic cases and contact them promptly. Measurement bias was minimized by using standardized PCR testing protocols.

### 2.6. Sample Size

The study utilized the entire population of HCWs participating in the surveillance system, providing a comprehensive sample to assess the COVID-19 infection rates and risk factors for severity. A total of 3100 HCWs were included in the study, contributing 7049 laboratory samples, as each HCW experienced between 1 and 14 diagnostic events during the study period.

### 2.7. Statistical Methods and Analysis

In the analysis of COVID-19 data among HCWs, multiple statistical approaches were employed to evaluate the distribution, characteristics, and severity of cases in alignment with the study objectives. The statistical analysis was designed to address five primary objectives, with a *p* < 0.05 considered as the threshold for statistical significance: (1) The distribution of COVID-19 cases across eight epidemiological waves: A descriptive analysis was performed to examine the frequency and proportion of suspected and confirmed COVID-19 cases in each wave, including an assessment of severe cases (hospitalizations and fatalities). This analysis provided insights into changes in disease burden over time and the impact of vaccination. (2) The sociodemographic characteristics of patients with and without COVID-19: A bivariate analysis explored differences in sociodemographic and clinical characteristics between positive and negative individuals with COVID-19. (3) Multivariable logistic regression model for sociodemographic and clinical characteristics associated with COVID-19: Repeated measures were used to control for covariance due to multiple events in the same HCW, and factors associated with COVID-19 were identified, adjusting for confounders, calculating odds ratios (ORs), confidence intervals (CIs.95%), and *p* value. (4) The frequency of symptoms and comorbidities in confirmed COVID-19 patients: Heatmaps were used to visualize the frequency of symptoms and comorbidities over time, identifying persistent factors such as hypertension and obesity across pandemic waves. (5) COVID-19 vaccination scheme across multiple doses: A Sankey diagram represents the progression of vaccine doses, highlighting variations in the type of vaccine and the number of individuals who did not complete the vaccination schedule. (6) Sociodemographic characteristics among severe and no-severe COVID-19 patients: Bivariate analysis compared severe (hospitalized or deceased) and non-severe cases, examining demographic and clinical variables such as sex, age, occupation, symptoms, comorbidities, and vaccination status; and (7) Multivariable logistic regression model for severity of COVID-19 outcomes: This model uses repeated measures to account for multiple events per HCW, controlling for covariance. In both, the factors associated with severe outcomes were identified by adjusting for sociodemographic and clinical characteristics. We used a *p* value cutoff of <0.25 for variable selection in the multivariable analysis, with sex included, regardless of its p value, as a key control variable. The analysis was performed in Stata version 15.0 by StataCorp LLC, College Station, TX, USA.

## 3. Results

We analyzed 7049 laboratory samples from healthcare workers (HCWs) from 2020 to 2024. Among the total sample, 4531 (64.28%) were females, and 2518 (35.72%) were males; the mean age was 39.91 years (standard deviation (SD) 11.27), with a range from 18 to 84 years. In terms of occupation, 2461 (34.92%) were nurses, and 1918 administrative personnel (27.21%)

### 3.1. Distribution of COVID-19 Cases Across Eight Epidemiological Waves

From March 2020 to May 2024, eight epidemiological waves were observed among HCWs, with a total of 7049 suspected cases, and 2838 (40.26%) positive cases; males (1063/2518, 42.22%) vs. females (1775/4531, 39.17%) and older age groups presented higher COVID-19 prevalence rates, indicating potential gender and age-related vulnerability.

During the study period, eight waves were identified: the majority of positive cases occurred in wave 5, with 777 cases; wave 4, with 664; and wave 2, with 415 cases. The highest positivity rates were identified in waves 5, 4 and 6, with 57.9%, 49.26% and 44.0%, respectively. Patients with confirmed cases of COVID-19 and the positivity index according to epidemiological waves are reported in Table 1.

**Figure 1:**
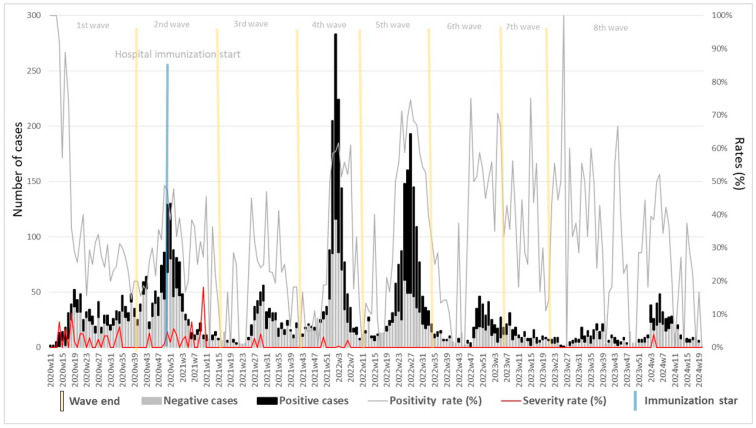
Temporal distribution of symptomatic healthcare workers (HCWs) by epidemiological week, from 2020–2024, with corresponding positivity and hospitalization rates. The figure illustrates the trend of negative and positive COVID-19 cases among HCWs, with the positivity rate (%) across multiple epidemic waves and the post-COVID-19 emergency period. The beginning of hospital vaccination by health personnel is also marked, providing context for the observed patterns. Blue line corresponds to vaccination in healthcare workers an yellow line corresponds to periods of epidemilogical waves.

All cases (n = 7049) were categorized into negative (black bar) and confirmed (gray bar) COVID-19 cases, with significant variation across each wave. In addition, severe cases (hospitalized) were identified throughout this period. Forty-six of the 48 severe cases occurred in the first four waves, mainly in waves 1 and 2, with 17 and 23 cases, respectively; only six hospitalized cases died during the study period, with three in the first wave and three in the second wave. This analysis provided information on the impact of vaccination on HCWs (Figure 1).

After the start of vaccination, particularly from the fifth wave onward, a substantial reduction in severe cases was observed, despite fluctuations in the number of confirmed cases. This trend underscores the protective impact of vaccination in reducing severe COVID-19 cases among HCWs, even as they return to routine activities and face ongoing exposure.

### 3.2. Sociodemographic Characteristics of Patients with and Without COVID-19

A bivariate analysis revealed differences between several characteristics of patients with and without COVID-19, and we observed differences in the distributions of sex, age groups, occupation, smoking, symptoms, diabetes and the number of vaccine doses. The distribution of cases according to occupation was as follows: nurses, 34.30%; administrative, 26.93%; and medical residents, 9.45%. On the other hand, the prevalence rates of COVID-19 according to occupation were 46.43% in paramedics, 44.6% in medical staff, 41.92% in personnel of general services, and 39.54% in nurses. The most frequent symptoms identified in confirmed cases of COVID-19 were cough (68.22%), rhinorrhea (44.29%), chills (43.38%), fever (36.75%), myalgias (33.65%), generalized attack (25.12%), and pharyngodynia (23.57%). With respect to comorbidities, we only observed differences in diabetes, with 7.72% in positive cases and 6.44% in negative cases. Vaccination appeared protective, with lower infection rates among those receiving two or more doses (Table 2).

Multivariable logistic regression analysis identified the sociodemographic and clinical risk factors associated with COVID-19 patients. Age was associated with an increased risk of infection in the older age groups > 70 years (OR = 5.71; 95% CI 2.08–15.63, *p* = 0.001), 60–69 years (OR = 2.25, 95% CI 1.5–3.37; *p* < 0.001), and 50–59 years (OR = 1.63, 95% CI 1.29–2.05, *p* < 0.001). Frontline healthcare workers had a greater risk of infection, indicating an elevated exposure risk: medical residents (OR = 2.79; 95% CI 2.08–3.74, *p* < 0.001), medical students (OR = 2.22; 95% CI 1.38–3.54; *p* = 0.001), and medical staff (OR = 2.21; 95% CI 1.63–2.97; *p* < 0.001). Individuals with symptoms such as chills, fever, anosmia, cough, diarrhea, and myalgias were more likely to test positive for COVID-19. In waves 1–3, the risk of developing COVID-19 was greater (OR = 16.06; 95% CI 10.40–24.81, *p* < 0.001), whereas in waves 4–6, the risk of developing COVID-19 was greater (OR = 11.65; 95% CI 9.23–14.70, *p* < 0.001). Vaccination had a protective effect, with those receiving two to three doses exhibiting a lower risk of contracting COVID-19 (OR = 0.67; 95% CI 0.51 to 0.89; *p* = 0.005) (Table 3).

### 3.3. Frequency of Symptoms and Underlying Comorbidities in Confirmed COVID-19 Patients

**Figure 2:**
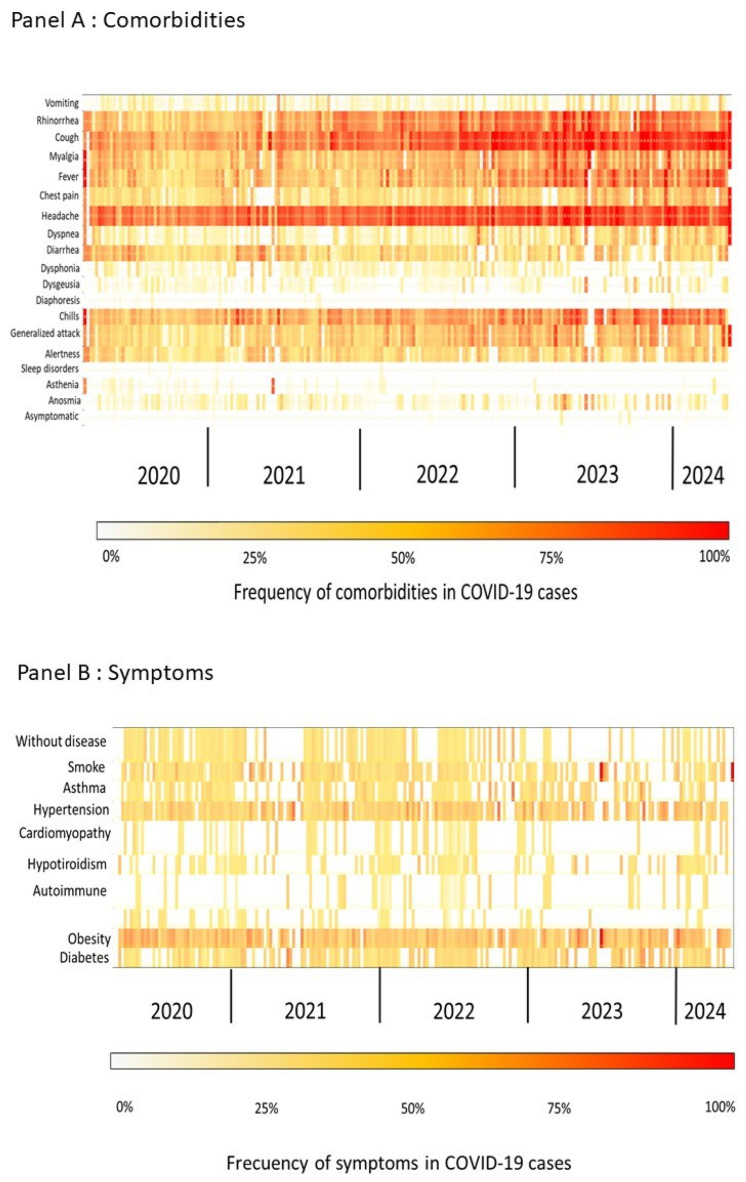
Heatmap of comorbidity and symptom frequency among healthcare workers during the COVID-19 pandemic from 2020 to 2024. This figure presents a heatmap illustrating the distribution and frequency of several comorbidities and symptoms reported by healthcare workers throughout the COVID-19 pandemic, spanning from 2020 to 2024. The intensity of color reflects the prevalence of specific conditions and symptoms over time.

A heatmap analysis of symptoms and underlying comorbidities in confirmed COVID-19 patients revealed temporal and intensity-based patterns over the study period. The frequency of symptoms, represented in a heatmap (Panel A from Figure 2), shows distinct patterns corresponding with epidemiological waves, with the highest intensity observed during peak periods of infection. Symptoms such as fever, cough, and fatigue were consistently prevalent across waves, with an increase in symptom intensity aligning with waves associated with a higher COVID-19 incidence, suggesting that the symptomatic burden intensified during times of elevated transmission (Figure 2).

In Panel B of Figure 2, the heatmap illustrates the frequency of symptoms and comorbidities among COVID-19-positive cases. Notably, rhinorrhea (range: 0–100%, mean: 61%) and headache (range: 0–100%, mean: 43%) persist as prevalent symptoms, particularly during early waves, highlighting their importance as frequent clinical manifestations. Other common symptoms include generalized attack (range: 0–100%, mean: 25%), fever (range: 0–100%, mean: 41%), cough (range: 0–100%, mean: 36%), and myalgia (range: 0–100%, mean: 32%), all of which were consistently observed, especially in the initial waves. Dyspnea (range: 0–100%, mean: 12%) was also notable, particularly in patients with respiratory complications.

Regarding comorbidities by week, hypertension (range: 0–50%, mean: 12%) and obesity (range: 0–100%, mean: 18%) were the most prevalent factors, particularly during the early waves, followed by diabetes (range: 0–43%, mean: 7%) and asthma (range: 0–50%, mean: 5%). These chronic conditions were frequently observed in COVID-19-positive patients and may have contributed to increased disease severity. Additionally, the percentage of patients without any comorbidities ranged from 0% to 25% (mean: 3%).

### 3.4. Immunization Scheme Across Vaccine Doses

**Figure 3:**
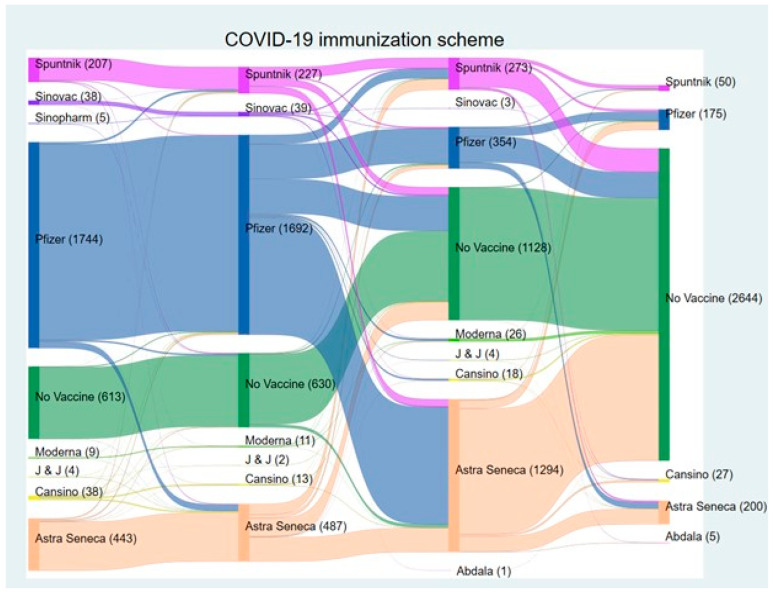
Sankey diagram illustrating the progression of self-reported COVID-19 immunization schemes by vaccine type from 2020 to 2024. This figure depicts the flow of healthcare workers at the NIC through various COVID-19 vaccine schemes over time. Each color path represents different vaccines, the transition between vaccine doses and combinations across different brands, highlighting the dynamic nature of vaccination strategies throughout the 2020–2024 period.

The Sankey diagram (Figure 3) illustrates the distribution and transition of COVID-19 vaccines administered from the first to the fourth dose during the pandemic. A considerable number of individuals remained unvaccinated throughout, with non-vaccination reaching 2644 by the fourth dose, indicating a substantial gap in vaccine uptake.

The Pfizer vaccine was the most frequently administered vaccine, with the first and second doses totaling 1744 doses and 1692, respectively, followed by AstraZeneca and Sputnik. The high initial reliance on Pfizer suggests its early availability. As the vaccination campaign progressed, AstraZeneca became the predominant vaccine for subsequent doses, with 1294 individuals receiving it as a third dose, likely due to supply shifts (Figure 3).

The transition patterns between doses show a decrease in continuity, with individuals switching to different vaccines for later doses. This shift, particularly toward AstraZeneca at the third dose, reflects adaptations in response to vaccine availability constraints (Figure 3).

### 3.5. Sociodemographic and Clinical Characteristics of Hospitalized COVID-19 Patients

In the bivariate analysis used to compare the sociodemographic and clinical characteristics of severe cases (hospitalized and deceased) with those of non-severe COVID-19 cases, no sex differences were observed; age was a relevant determinant, and 40 severe cases were identified in the 40–49 years and older age groups, highlighting age as a risk factor for adverse outcomes. The distribution by occupation differed between COVID-19 cases and severe cases (*p* = 0.003). According to the eight waves analyzed in this study, cases requiring hospitalization occurred mainly in the first wave, with 17 (35.42%) cases in the first wave, 23 (42.97%) in the second wave, and 3 (6.25%) cases during waves 3 and 4, *p* < 0.001. The main symptoms in severe cases of COVID-19 were cough (70.8%), fever (62.5%), chills (54.2%), myalgias (37.5%), and arthralgias (34.5%). Additionally, dyspnea was observed in 25.0% of patients. The underlying comorbidities most common in hospitalized patients were hypertension (37.5%), obesity (35.42%), diabetes mellitus (14.58%), and cardiopathy (10.42%), which were more common among severe patients, underscoring the impact of chronic conditions on COVID-19 progression. Vaccination status also played a significant role; of the 48 severe cases, 40 (83.33%) that occurred in the first wave were in people who were not vaccinated (Table 4).

### 3.6. Multivariable Analysis of Sociodemographic Characteristics and Severity of COVID-19

A multivariable logistic regression model assessing the factors associated with severe COVID-19 outcomes (hospitalization or death) among confirmed cases revealed that male patients did not show a statistically significant difference in severity compared with female patients (OR = 1.0; 95% CI 0.49–2.65; *p* = 0.987). Age was a relevant characteristic, with individuals aged >70 years having the greatest association with disease severity (OR = 14.09, 95% CI 1.21–163.87; *p* = 0.035), alongside those aged 60–69 years (OR = 6.98, 95% CI 1.53–31.71, *p* = 0.012), and those aged 40–49 years (OR = 3.72 (95% CI 1.2–11.46; *p* = 0.022). Occupational differences were observed; although not statistically significant, medical residents had a greater likelihood of experiencing severe outcomes (OR = 4.09; 95% CI 0.83–19.97; *p* = 0.082), alongside medical staff (OR = 2.77; 95% CI 0.16–43.8340; *p* value = 0.311). Notably, patients diagnosed during waves 1 to 3 (OR = 1.11; 95% CI 1.12–110.21; *p* = 0.04) had a significantly greater risk of severe outcomes than those diagnosed during waves 6–8. (Table 5). Individuals with zero to one dose of vaccine had a greater risk of severe outcomes (OR = 2.7; 95% CI 0.16 to 43.83, *p* = 0.484), and those with two to three doses of vaccine (OR = 1.1, 95% CI 0.11 to 0.74; *p* = 0.931) considered those with four doses as a reference group. Symptom presentation was associated with severity; individuals reporting fever had a higher likelihood of severe outcomes (OR = 6.63; 95% CI 1.91–6.88; *p* < 0.001). Comorbidities also played a significant role; obesity (OR = 2.33; 95% CI 1.17–4.63; *p* = 0.015) and hypertension (OR = 2.61; 95% CI 1.28–5.33; *p* = 0.008) were both associated with an increased risk of severe COVID-19. Diabetes was not significantly associated with disease severity in this model (OR = 1.61; 95% CI 0.62–4.12; *p* = 0.321) (Table 5).

## 4. Discussion

This study highlights the critical role of vaccination in preventing severe COVID-19 outcomes, such as hospitalizations and deaths, among healthcare workers (HCWs). The findings demonstrate the consistent protective effect of vaccines across multiple epidemiological waves in a high-risk healthcare setting, emphasizing the importance of vaccination in reducing the burden of severe disease in a vulnerable population. However, an important insight is that vaccines, particularly in the context of a highly transmissible virus such as SARS-CoV-2, do not prevent infection entirely; rather, they significantly reduce disease severity. This misconception—that vaccines entirely prevent COVID-19 infections—has led to a false sense of security among some vaccinated individuals, resulting in continued transmission [5,6,16,17,27].

The association between vaccination adherence and reduced severity aligns with the literature supporting vaccine effectiveness in high-risk populations. While our results confirm that severe cases decreased significantly following the second dose, they also highlight the role of vaccination in transitioning COVID-19 from a public health emergency toward endemicity. Existing theories on endemicity suggest that reduced case severity, in conjunction with widespread immunity from vaccination and prior infection, can help stabilize infection rates [15,22,23,26,27,28]. However, this transition requires ongoing epidemiological and genomic surveillance, as emerging variants may challenge existing immunity, necessitating further vaccine updates or booster programs. The data suggest that vaccination alone may not sustain endemic stability; it must be accompanied by surveillance for new variants to maintain control over potential future outbreaks.

The study also provides valuable insights into the real-world impact of vaccination on HCWs—a group with substantial exposure risks. Vaccination campaigns initially prioritized HCWs, recognizing the importance of protecting those at the frontline of care. The prioritization of HCWs in vaccination efforts has proven effective; despite continuous exposure, infection rates remain manageable, underscoring the strategic wisdom of early vaccination for HCWs [15,17,27].

The Sankey diagram (Figure 3) illustrates the dynamic nature of vaccination uptake among HCWs throughout the pandemic. It highlights several key patterns, including the substantial number of individuals who remained unvaccinated, which grew to 2644 by the fourth dose. This trend underscores the persistent challenge of vaccine hesitancy or logistical barriers that may have prevented individuals from completing their vaccination series. Transition patterns between doses reveal shifts in vaccine type, such as the early dominance of Pfizer in the first and second doses (1744 and 1692 doses, respectively) and the subsequent predominance of AstraZeneca for the third dose (1294 doses). These variations could reflect changes in vaccine availability or procurement policies during the pandemic. Such transitions, while necessary, may have contributed to vaccine hesitancy, as individuals might have been reluctant to mix vaccine types or expressed preferences for specific vaccines technologies, mainly in vaccination reinforcement doses.

Additionally, vaccine adherence among HCWs is influenced by a complex interplay among logistical, perceptual, and systemic factors [5,14,31]. The significant drop in vaccination rates observed between doses, as depicted in the Sankey diagram, could reflect both structural barriers, such as vaccine availability and access, and psychological barriers, including vaccine hesitancy driven by misinformation or concerns about safety and efficacy. Furthermore, evolving risk perceptions, particularly as the perceived severity of COVID-19 decreased over time, may have contributed to the reduced urgency of subsequent doses [14,23,32,33,34]. Addressing these challenges requires a multifaceted approach, combining targeted education to counter misinformation, logistical strategies to ensure consistent vaccine availability, and policy interventions that incentivize vaccine adherence among HCWs. By identifying and mitigating these barriers, healthcare systems can optimize vaccine uptake, ultimately enhancing the resilience and preparedness of HCWs in future health crises.

In Mexico, a study conducted in northeastern regions revealed that 5.5% of healthcare workers expressed vaccine hesitancy, with misinformation identified as the primary determinant for rejecting vaccination. This emphasizes the importance of implementing targeted educational strategies to address misinformation and improve vaccine acceptance among health personnel [33]. Globally, COVID-19 vaccine hesitancy among HCWs varied significantly, ranging from 4.3% to 72%, with an average hesitancy rate of 22.51% [14]. Demographic factors, such as being male, older, or having higher education levels, were consistently associated with greater vaccine acceptance, whereas concerns about safety and side effects were the primary reasons for hesitancy [14]. Studies in Arab countries further highlighted moderate acceptance rates, with significant variation by gender and risk perception [34]. These illustrate the shared global challenges associated with addressing vaccine hesitancy among HCWs while highlighting regional variations in the factors influencing acceptance.

Our results suggest that vaccines are not solely preventive tools but also essential measures to sustain health service operations by protecting those at the highest risk. Furthermore, these findings underscore the need for ongoing vaccination advocacy and education, emphasizing the safety and efficacy of vaccines, particularly in emergency contexts, to maintain high adherence and reinforce public confidence in vaccination.

Comorbidities and underlying conditions increase the risk of severe outcomes, a pattern evident throughout the pandemic. While these conditions initially increased the risk of severe COVID-19 outcomes, subsequent vaccinations significantly reduced this risk. The initial vulnerability observed in unvaccinated individuals with comorbidities shifted as both vaccination and subsequent infections decreased in severity over time. This moderating effect highlights the protective synergy between vaccination and natural immunity over repeated exposures, which contributes to a downward trend in severe cases [17,22,23,26,27]. By addressing these risk factors through vaccination and ongoing surveillance, healthcare systems can better anticipate which populations may still require additional protection or tailored interventions [16,17,29].

Our study’s findings carry broader implications for public health. Vaccination remains an essential tool in infection control, yet it must be paired with robust surveillance systems to monitor infection trends, respond to emerging risks, and adapt public health strategies as needed [15,17,23,26,27,28]. For HCWs, particularly those with heightened susceptibility due to preexisting conditions, an integrated approach comprising vaccination and active surveillance provides a comprehensive framework to protect both individuals and the healthcare infrastructure. Individuals who were not vaccinated were more likely to develop severe disease, reflecting the protective effect of increased vaccine coverage. Additionally, those with a history of prior COVID-19 infections tended to have lower severity rates, possibly due to acquired immunity. Maintaining workforce health through these measures is critical for preventing system overload during pandemic surges, ensuring that healthcare services remain resilient and prepared for future infectious threats [15,26,27].

Future research should prioritize longitudinal studies to examine vaccine efficacy over time, particularly in the context of emerging variants that may challenge established immunity. Studies focusing on vaccine effectiveness within various demographic and comorbidity profiles will enhance our understanding of how different groups respond to vaccines over time, helping to develop more tailored protective strategies. Moreover, examining societal perceptions of diverse vaccine types and understanding the factors influencing vaccine hesitancy will be instrumental in designing effective vaccination campaigns. Investigating the real-world interactions of vaccines with new SARS-CoV-2 variants will also be crucial for sustaining endemicity, as such findings will inform the development of adaptable vaccine strategies for HCWs and the broader population.

## 5. Limitations

Our limitations include the potential for bias related to self-reported symptoms and comorbidity data, and the generalizability of findings beyond the study setting. As a single-institution study, the findings reflect the experiences and exposures specific to the setting, limiting direct application to other contexts. Furthermore, data completeness, particularly for vaccination adherence over time, may impact the broader interpretation of vaccine effectiveness across multiple doses. These limitations, while inherent in the study design, underscore the need for the cautious interpretation of the results.

## 6. Conclusions

The broader impact of this study lies in its reinforcement of vaccination as one of the main tools for infection control in healthcare settings. These findings support the integration of targeted vaccination campaigns tailored to HCWs, addressing barriers to multidose adherence to maintain immunity levels across waves. Furthermore, this research provides valuable insights into infection trends among HCWs, underscoring the importance of surveillance systems to monitor and respond to evolving risks within healthcare settings.

Future research should prioritize longitudinal studies that track vaccine efficacy over time and across successive doses to better understand the duration of immunity in healthcare environments. Additionally, examining the interaction between specific comorbidities and vaccination status will further elucidate the risk factors associated with severe COVID-19 outcomes, enhancing protective strategies. Studies assessing emerging vaccines and their effectiveness against novel variants will also be valuable for ongoing infectious disease preparedness [3,15,17,22].

## Figures and Tables

**Table 1:** Distribution of positive and negative cases of COVID-19 according to wave from 2020 to 2024.

Wave or Period	COVID-19			
	Negative n = 4211 n (%)	Positive n = 2838 n (%)	Total n = 7049 n (%)	Positivity Rate (%)
**Wave 1** (15 March to 29 September 2020)	561 (13.32)	270 (9.51)	831 (11.79)	32.49
**Wave 2** (30 September 2020 to 15 April 2021)	824 (19.57)	415 (14.62)	1239 (17.58)	33.49
**Wave 3** (16 April to 21 October 2021)	407 (9.67)	127 (4.47)	534 (7.58)	23.78
**Wave 4** (22 October 2021 to 4 March 2022)	684 (16.24)	664 (23.40)	1348 (19.12)	49.26
**Wave 5** (5 March to 19 August 2022)	584 (13.87)	777 (27.38)	1361 (19.31)	57.09
**Wave 6** (20 August 2022 to 4 February 2023)	238 (5.65)	187 (6.59)	425 (6.03)	44.00
**Wave 7** (5 February to 13 May 2023)	119 (2.83)	76 (2.68)	195 (2.77)	38.97
**Post-COVID-19 Emergency** (14 May 2023 to 20 May 2024)	794 (18.86)	322 (11.35)	1116 (15.83)	28.85
**Total**	**4211**	**2838**	**7049**	**40.26**

Totals are presented in columns. Positivity rates were calculated for each wave.

**Table 2:** Characteristics of healthcare workers (HCWs) tested for COVID-19 from 2020 to 2024.

Variable	COVID-19		Total n = 7049 n (%)	*p* Value
	Negative n = 4211 n (%)	Positive n = 2838 n (%)		
Sex				
Female	2756 (65.45)	1775 (62.54)	4531 (64.28)	0.013
Male	1455 (34.55)	1063 (37.46)	2518 (35.72)	
Age group				
18 to 29	1003 (23.82)	611 (21.53)	1614 (22.90)	<0.001
30 to 39	1198 (28.45)	721 (25.41)	1919 (27.22)	
40 to 49	1177 (27.95)	816 (28.75)	1993 (28.27)	
50 to 59	691 (16.41)	531 (18.71)	1222 (17.34)	
60 to 69	132 (3.13)	137 (4.83)	269 (3.82)	
70 years	10 (0.24)	22 (0.78)	32 (0.45)	
Occupation				
Nurse	1488 (35.34)	973 (34.30)	2461 (34.92)	0.003
Medical resident	438 (10.40)	268 (9.45)	706 (10.02	
Medical student	147 (3.49)	68 (2.40)	215(3.05)	
Medical staff	311 (7.39)	245 (8.64)	556 (7.89)	
Administrative	1154 (27.40)	764 (26.93)	1918 (27.21)	
General services	248 (5.89)	179 (6.31)	427 (6.06)	
Paramedics	300 (7.12)	260 (9.16)	560 (7.95)	
Researchers	125 (2.97)	80 (2.82)	205 (2.91)	
Total				
Clinical and epidemiological characteristics				
Smoking	303 (7.19)	163 (5.74)	466 (6.61)	0.071
Signs and symptoms *				
Cough	1796 (42.65)	1936 (68.22)	3732 (52.94)	<0.001
Rhinorrhea	1391 (33.03)	1257 (44.29)	2648 (37.57)	<0.001
Chills	1244 (29.54)	1231 (43.38)	2475 (35.11)	<0.001
Fever	922 (21.90)	1043 (36.75)	1965 (27.88)	<0.001
Myalgia	964 (22.89)	955 (33.65)	1919 (27.22)	<0.001
Generalized Attack	753 (17.88)	713 (25.12)	1466 (20.80)	<0.001
Pharyngodynia	883 (20.97)	669 (23.57)	1552 (22.02)	0.011
Arthralgia	724 (17.19)	707 (24.91)	1431 (20.30)	<0.001
Diarrhea	794 (18.86)	470 (16.56)	1264 (17.93)	0.014
Alertness	684 (16.24)	541 (19.06)	1225 (17.38)	0.002
Chest Pain	638 (15.15)	556 (19.59)	1194 (16.94)	<0.001
Abdominal Pain	517 (12.28)	321 (11.31)	838 (11.89)	0.219
Dyspnea	335 (7.96)	256 (9.02)	591 (8.38)	0.114
Dysphonia	229 (5.44)	145 (5.11)	374 (5.31)	0.546
Vomiting	201 (4.77)	120 (4.23)	321 (4.55)	0.282
Dysgeusia	94 (2.23)	138 (4.86)	232 (3.29)	<0.001
Comorbidities *				
Obesity	736 (17.48)	483 (17.02)	1219 (17.29)	0.617
Hypertension	504 (11.97)	380 (13.39)	884 (12.54)	0.077
Diabetes Mellitus	271 (6.44)	219 (7.72)	490 (6.95)	0.038
Asthma	191 (4.54)	111 (3.91)	302 (4.28)	0.204
Cardiopathy	58 (1.38)	44 (1.55)	102 (1.45)	0.551
Number of COVID-19 vaccine doses				
None	1415 (33.60)	703 (24.77)	2118 (30.05)	<0.001
1st dose	7 (0.17)	13 (0.46)	20 (0.28)	
2nd dose	1114 (26.45)	490 (17.27)	1604 (22.76)	
3rd dose	1368 (32.49)	1377 (48.52)	2745 (38.94)	
4th dose	307 (7.29)	255 (8.99)	562 (7.97)	

Multivariable analysis of sociodemographic characteristics and COVID-19 and non-COVID-19 patients. * Each variable is considered as a separate characteristic.

**Table 3:** Multivariable logistic regression model evaluating risk factors for healthcare workers (HCWs) developing COVID-19.

Variable/Category	Odds Ratio (OR)	95%CI Lower	95%CI Upper	*p* Value
Sex				
Females	Reference category			
Males	0.99	0.84	1.18	0.990
Age group				
18 to 29	1.56	1.25	1.94	<0.001
40 to 49	1.22	0.99	1.50	0.058
50 to 59	1.63	1.29	2.05	<0.001
60 to 69	2.25	1.50	3.37	<0.001
>70 years	5.71	2.08	15.63	0.001
Previous COVID-19 infections				
Yes	26.95	21.88	33.20	<0.001
Health care workers (HCW)				
Nurse	Reference category			
Medical resident	2.79	2.08	3.74	<0.001
Medical student	2.22	1.38	3.54	0.001
Medical staff	2.21	1.63	2.97	<0.001
Administratives	1.11	0.91	1.34	0.300
General services	1.32	0.94	1.84	0.104
Paramedics	1.62	1.21	2.17	0.001
Reserchers	1.96	1.25	3.09	0.003
Vaccine periods				
Wave 1 to 3	16.06	10.40	24.81	<0.001
Wave 4 to 6	11.65	9.23	14.70	<0.001
Wave 7 to Post-COVID-19 Emergency	Reference category			
Self-reported symptomatology				
Chills	1.20	1.01	1.42	0.032
Fever	1.75	1.47	2.10	<0.001
Anosmya	1.56	1.10	2.22	0.011
Cough	2.42	2.07	2.83	<0.001
Diahrrea	0.71	0.59	0.86	<0.001
Mialgya	1.30	1.09	1.54	0.003
Self-reported Comorbidities				
Obesity	0.81	0.66	0.97	0.026
Immunization scheme				
0 to 1	1.01	0.64	1.58	0.978
2 to 3	0.67	0.51	0.89	0.005
4 doses	Reference category			

Variables included in the table, such as age, sex, professional role, previous COVID-19 infections, self-reported symptomatology, and comorbidities, were used as adjustment variables. These adjustment variables were selected to control for confounding factors and provide a comprehensive analysis of the risk factors associated with COVID-19 among HCWs.

**Table 4:** Characteristics of healthcare workers (HCWs) with severe COVID-19 from 2020 to 2024.

Variable	Non-Severe COVID-19 n = 2790	Severe COVID-19 n = 48	Total n = 2838	*p* Value
**Sex**				
Female	1748 (62.65)	27 (56.25)	1775 (62.54)	0.364
Male	1042 (37.35)	21 (43.75)	1063 (37.46)	
**Age group**				
18 to 29	609 (21.83)	2 (4.17)	611 (21.53)	0.001
30 to 39	715 (25.63)	6 (12.50)	721 (25.41)	
40 to 49	795 (28.49)	21 (43.75)	816 (28.75)	
50 to 59	518 (18.57)	13 (27.08)	531 (18.71)	
60 to 69	132 (4.73)	5 (10.42)	137 (4.83)	
70 to 90	21 (0.75)	1 (2.08)	22 (0.78)	
**Occupation**				
Nurse	960 (34.42)	13 (27.08)	973 (34.30)	0.03
Medical resident	265 (9.50)	3 (6.25)	268 (9.45)	
Medical student	68 (2.44)	0 (0.00)	68 (2.40)	
Medical staff	238 (8.53)	7 (14.58)	245 (8.64)	
Administratives	744 (26.68)	20 (41.67)	764 (26.93)	
General services	174 (6.24)	5 (10.42)	179 (6.31)	
Paramedics	260 (9.32)	0 (0.00)	260 (9.16)	
Researchers	80 (2.87)	0 (0.00)	80 (2.82)	
**Clinical and pidemiological characteristics**				
Smoking	170 (6.09)	3 (6.25)	173 (6.10)	0.964
**Epidemiological wave**				
Wave 1	253 (9.07)	17 (35.42)	270 (9.51)	<0.001
Wave 2	392 (14.05)	23 (47.92)	415 (14.62)	
Wave 3	124 (4.44)	3 (6.25)	127 (4.47)	
Wave 4	661 (23.69)	3 (6.25)	664 (23.40)	
Wave 5	777 (27.85)	0 (0.00)	777 (27.38)	
Wave 6	187 (6.70)	0 (0.00)	187 (6.59)	
Wave 7	76 (2.72)	0 (0.00)	76 (2.68)	
Wave 8	320 (11.47)	2 (4.17)	322 (11.35)	
**Signs and symptoms**				
Cough	1902 (68.17)	34 (70.83)	1936 (68.22)	0.695
Rhinorrhea	1242 (44.52)	15 (31.25)	1257 (44.29)	0.067
Chills	1205 (43.19)	26 (54.17)	1231 (43.38)	0.128
Fever	1013 (36.31)	30 (62.50)	1043 (36.75)	<0.001
Myalgias	937 (33.58)	18 (37.50)	955 (33.65)	0.569
Generalized Attack	699 (25.05)	14 (29.17)	713 (25.12)	0.515
Arthralgias	690 (23.73)	17 (35.42)	707 (24.91)	0.139
Dyspnea	244 (8.75)	12 (25.0)	256 (9.02)	<0.001
Pharyngodynia	662 (23.73)	7 (14.58)	669 (23.57)	0.139
Chest Pain	549 (19.68)	7 (14.58)	556 (19.59)	0.378
Alertness	535 (19.18)	6 (12.50)	541 (19.06)	0.243
Diarrhea	456 (16.34)	14 (29.17)	470 (16.56)	0.018
Vomiting	115 (4.12)	5 (10.42)	120 (4.23)	0.032
Abdominal Pain	317 (11.36)	4 (8.33)	321 (11.31)	0.511
Dysphonia	144 (5.16)	1 (2.08)	145 (5.11)	0.337
Dysgeusia	137 (4.91)	1 (2.08)	138 (4.86)	0.367
**Comorbidities**				
Obesity	466 (16.70)	17 (35.42)	483 (17.02)	0.001
Hypertension	362 (12.97)	18 (37.50)	380 (13.39)	<0.001
Diabetes Mellitus	212 (7.60)	7 (14.58)	219 (7.72)	0.072
Asthma	110 (3.94)	1 (2.08)	111 (3.91)	0.51
Cardiopathy	39 (1.40)	5 (10.42)	44 (1.55)	<0.001
**Number of COVID-19 vaccine dosis**				
None	663 (23.76)	40 (83.33)	703 (24.77)	<0.001
1st dose	13 (0.47)	0 (0.00)	13 (0.46)	<0.001
2nd dose	484 (17.35)	6 (12.50)	490 (17.27)	<0.001
3rd dose	1376 (49.32)	1 (2.08)	1377 (48.52)	<0.001
4th dose	254 (9.10)	1 (2.08)	255 (8.99)	<0.001

**Table 5:** Multivariable logistic regression model evaluating the risk factors for HCWs developing severe COVID-19.

Variable/Category	Odds Ratio (OR)	95%CI Lower	95%CI Upper	*p* Value
Sex				
Male	1.0	0.49	2.03	0.987
Age group				
18 to 29	0.51	0.09	2.65	0.427
30 to 39	Reference category			
40 to 49	3.72	1.2	11.46	0.022
50 to 59	3.44	1.02	11.6	0.046
60 to 69	6.98	1.53	31.71	0.012
>70 years	14.09	1.21	163.87	0.035
Health care workers (HCW)				
Nurse	Reference category			
Medical resident	4.09	0.83	19.97	0.082
Medical student	1.0	(empty) *	(empty) *	(empty) *
Medical doctor	1.77	0.58	5.4	0.311
Administratives	1.61	0.72	3.59	0.236
General services	1.12	0.32	3.88	0.855
Paramedics	1	(empty) *	(empty) *	(empty) *
Reserchers and academics	1	(empty) *	(empty) *	(empty) *
Epidemiological waves				
Wave 1 to 3	11.11	1.12	110.21	0.040
Wave 4 to 6	1.28	0.22	7.2	0.778
Wave 7 to Post-COVID-19 Emergency	Reference category			
Self-reported symtomatology				
Fever	6.63	1.91	6.88	<0.001
Self-reported Comorbidities				
Obesity	2.33	1.17	4.63	0.015
Hypertension	2.61	1.28	5.33	0.008
Diabetes	1.61	0.62	4.12	0.321
Immunization scheme				
0 to 1	2.70	0.16	43.83	0.484
2 to 3	1.11	0.11	10.74	0.931
4 doses	Reference category			

Odds ratios (ORs), 95% confidence intervals (CIs), and *p* values from the Wald test. Footnote: * (empty) indicates that the variable perfectly predicts the outcome in this subset of the data, resulting in its exclusion from the analysis for these specific cases. However, the variable remains in the model because it provides valuable information for other subsets of the data where its inclusion does not lead to perfect prediction.

## Data Availability

The dataset is available upon request from the authors.
